# A Novel Data-Mining Approach Systematically Links Genes to Traits

**DOI:** 10.1371/journal.pbio.0030166

**Published:** 2005-04-05

With exponential advances in computing power over the past ten years, data-generating capacity has far outpaced anyone's ability to mine the rich seams of information. This is especially true in the field of genomics. So far, over 222 prokaryote (bacteria) genomes have been sequenced, 21 archaea (primitive bacteria-like extremophiles), and 17 eukaryotes (from yeast to fly and rat to human), according to the Center for Biological Sequence Analysis in Denmark (http://www.cbs.dtu.dk/services/GenomeAtlas/). All these genomes promise to provide powerful insights into the biological processes of life, but such insights come with painstaking analysis by trained experts. Matching genotype to phenotype—the visible or measurable characteristics of species—is a major challenge in what Francis Collins, Director of the United States National Human Genome Research Institute, has called the “post-genomic era.”

In a new study, Peer Bork and a team of bioinformatics-savvy molecular biologists tested a new approach to extracting biologically meaningful information from the massive MEDLINE database. The US National Library of Medicine's MEDLINE contains over 12 million abstracts from thousands of publications dating back to 1965. Combining automated literature mining with comparative genomics—which compares genome sequences of different organisms to discern differences and similarities in gene content—the authors conducted a systematic search for associations between genes and phenotypic traits. Their approach automates tasks that typically require human curation.

Recognizing that the best source of information on species phenotypic traits is the scientific literature where biologists describe them, the authors first ran a search to identify associations between species and traits in MEDLINE abstracts. Words that tended to occur with subsets of species, the authors reasoned, were more likely to reflect particular traits. From a total of 255,249 MEDLINE abstracts showing any connection to 92 prokaryotic species with sequenced genomes, 172,967 nouns showed meaningful associations related to the species' traits. “Flagellum” and “motility” showed up more often in self-propelling species, for example, and “endosymbiont” aptly appeared with the intracellular bacteria (Buchnera aphidicola) that inhabits aphids.

Next, Bork and colleagues detected the presence or absence of over 200,000 evolutionarily conserved genes across the 92 species and sorted the results into species–word and species–gene groups. The analysis revealed a number of words and genes with similar distribution in related species, leading to over 2,700 significant associations between trait-descriptive words and orthologous (evolved from a common ancestor) groups of genes. These genes encode over 28,000 proteins. Many were already known—including genes involved in pathogenicity, biodegradation and biosynthesis, and photosynthesis—but many, the authors note, are “novel” or of “unexpected character and complexity.”

And it is the ability to uncover unexpected relationships across numerous genes and genomes—patterns likely to escape human analysis—that makes this approach so powerful. Among these unexpected match-ups, Bork and colleagues linked a number of food and food-poisoning-related terms with metabolic-enzyme-coding genes. All 37 genes predicted to play a role in food spoilage and toxicity are present in food-borne pathogens but not in most other prokaryotes. By assigning functions to these previously uncharacterized genes, the authors could also assign new roles for pathways that use the genes. For example, by linking two genes with pathways that metabolize propanediol and ethanolamine—compounds found almost exclusively in highly hazardous food-borne pathogens—the authors predict that propanediol and ethanolamine pathways are “crucial genomic determinants of pathogenicity associated with food poisoning.”[Fig pbio-0030166-g001]

**Figure pbio-0030166-g001:**
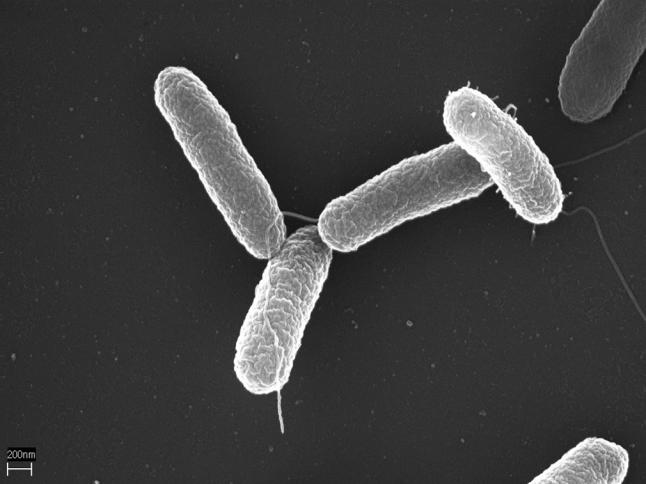
Many predicted genes were tied to food poisoning and bacterial pathogens, such as Salmonella typhimurium (Photo: Volker Brinkmann, Max Planck Institute for Infection Biology, Berlin, Germany)

That their analysis linked so many predicted genes with bacterial pathogenicity might be expected, the authors note, since both genome sequencing and biological research are heavily focused on human health. Given the weekly increase in the number of genomes sequenced and in MEDLINE entries, the method outlined here should provide a valuable tool to help researchers narrow the gap between the promise and payoff of the genomic revolution.

